# A multifunctional small RNA binding protein for sensing and signaling cell envelope precursor availability in bacteria

**DOI:** 10.15698/mic2020.05.717

**Published:** 2020-04-15

**Authors:** Muna A. Khan, Boris Görke

**Affiliations:** 1 Department of Microbiology, Immunobiology and Genetics, Max Perutz Labs, University of Vienna, Vienna Biocenter (VBC), 1030 Vienna, Austria.

**Keywords:** RNA binding protein, metabolite sensing, small regulatory RNA, two-component system, cell envelope precursor, Escherichia coli

## Abstract

Synthesis of glucosamine-6-phosphate (GlcN6P) by the enzyme GlmS initiates bacterial cell envelope biosynthesis. To ensure ongoing synthesis, GlcN6P homeostasis is required. *Escherichia coli* achieves this through a post-transcriptional control mechanism comprising the RNA-binding protein RapZ and small RNAs (sRNAs) GlmY and GlmZ. GlmZ stimulates *glmS* translation by base-pairing. When GlcN6P is abundant, GlmZ is cleaved and inactivated by endoribonuclease RNase E. Cleavage depends on RapZ, which binds GlmZ and recruits RNase E. Decreasing GlcN6P concentrations provoke up-regulation of the decoy sRNA GlmY which sequesters RapZ, thereby suppressing GlmZ decay. In our current study we identify RapZ as the GlcN6P sensor. GlcN6P-free RapZ interacts with and stimulates phosphorylation of the two-component system (TCS) QseE/QseF triggering *glmY* expression. Thereby generated GlmY sequesters RapZ into stable complexes, allowing for *glmS* expression. Sequestration by GlmY also disables RapZ to stimulate QseE/QseF, providing a negative feed-back loop limiting the response. When GlcN6P is replenished, GlmY is released from RapZ and rapidly degraded. Our work has revealed a complex regulatory scenario, in which an RNA binding protein senses a metabolite and communicates with two sRNAs, a TCS and ribonuclease RNase E to achieve metabolite homeostasis.

In recent years, post-transcriptional regulation exerted by sRNAs and RNA-binding proteins (RBPs) emerged as crucial layer for regulation of cellular activities in all organisms. However, only a handful of such RBPs have been characterized in bacteria, leaving important questions in the field unsolved. Traditionally, it is thought that RBPs control RNA fates but how they themselves are controlled and whether this could also involve RNA is largely unclear. It is also unknown how and to what extent RBPs are embedded in the protein-protein interaction network to possibly form higher-order complexes and whether there is direct cross-talk with transcriptional regulators.

In our recent study, we addressed these questions for the RBP RapZ in *E. coli*. Previous work has shown that RapZ acts in concert with two homologous sRNAs GlmY and GlmZ to achieve homeostasis of the key metabolite GlcN6P. All amino sugar constituents of the bacterial cell envelope, namely peptidoglycan and outer membrane lipopolysaccharides, derive from GlcN6P. The need for GlcN6P synthesis may change during the life-cycle of a bacterium depending on growth rate, extracellular amino sugar levels and presence of GlmS inhibitory antimicrobials produced by competing microorganisms. Therefore, bacteria must sense intracellular GlcN6P and adjust GlmS activity accordingly. To this end, many species rely on post-transcriptional mechanisms. In *E. coli*, the ribosomal binding site of the *glmS* mRNA is masked by an inhibitory stem loop structure. Assisted by the RNA chaperone Hfq, sRNA GlmZ base-pairs with the *glmS* mRNA and resolves this structure. Consequently, translation occurs, which concomitantly protects the mRNA from degradation **(Fig. 1)**. GlmZ itself is controlled at the level of decay. The endoribonuclease RNase E inactivates GlmZ by cleaving within its base-pairing site **(Fig. 1**, left). However, RNase E cannot act alone on GlmZ. Processing requires RapZ - the **R**Nase E **a**daptor **p**rotein for cleavage of Glm**Z**. RapZ interacts with RNase E and also binds GlmZ at its central stem loop using a C-terminal non-canonical RNA binding domain enriched in positively charged residues. RapZ, a tetramer, is envisioned to form an encounter complex with tetrameric RNase E to cleave the sandwiched sRNA. Under GlcN6P sufficiency, the majority of GlmZ molecules undergoes cleavage by the RapZ/RNase E complex, resulting in basal GlmS levels **(Fig. 1**, left). This basal level is adjusted by an inhibitory feed-back loop mediated by the processed GlmZ variant, which retains the ability to bind RapZ. When processed GlmZ accumulates, it sequesters RapZ at least partially, reducing ongoing GlmZ processing **(Fig. 1**, left). Upon GlcN6P scarcity, a second sRNA enters the game: GlmY is homologous to GlmZ, but lacks the *glmS* base-pairing site. Moreover, GlmY has low affinity for Hfq as Hfq-binding motifs are lacking or sequestered by secondary structure. Accordingly, GlmY does not act via base-pairing but through sequestration of protein RapZ. A processed variant of GlmY, generated by a yet unknown activity, specifically accumulates when GlcN6P concentrations decrease and sequesters RapZ through molecular mimicry **(Fig. 1**, right). Consequently, GlmZ stays intact leading to increased GlmS amounts that replenish GlcN6P. Experimentally, this cascade can be induced by antibiotics targeting GlmS, providing a tool for analysis. For instance, Nva-FMDP – a derivative of a dipeptide produced by *Streptomyces collinus* - binds and inhibits GlmS, blocking GlcN6P production. As a result, GlmY accumulates and increases GlmS synthesis through GlmZ. Interestingly, higher GlmS levels overcome inhibition by the drug, providing intrinsic resistance. Thus, the need for defense against antibiotics released by competing microorganisms may provide one explanation why many bacteria including *E. coli* feedback-regulate GlmS at the level of enzyme synthesis and not activity as observed in eukaryotes.

**Figure 1:**
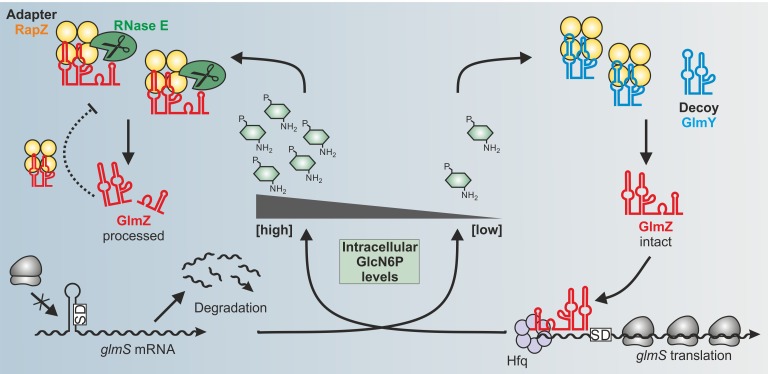
FIGURE 1: Model for the control of *glmS* mRNA translation by the GlmY/RapZ/GlmZ circuit in response to GlcN6P sufficiency (left) and starvation (right) conditions.

Until recently, it remained mysterious how the GlcN6P signal is sensed and processed by the GlmY/RapZ/GlmZ circuit. As *glmY* mutants fail to activate *glmS* expression under GlcN6P depletion, we initially hypothesized that GlmY itself or a factor upstream might sense this metabolite. Contrarily, our recent study revealed that in fact RapZ represents the sought GlcN6P sensor. RapZ binds GlcN6P *in vivo* as shown by targeted metabolomics, in which GlcN6P co-eluted with RapZ purified by affinity chromatography. Surface plasmon resonance spectroscopy (SPR) demonstrated specific and high affinity binding *in vitro*. Decreasing GlcN6P concentrations trigger incremental *glmY* transcription rates and *rapZ* mutants fail to do so. These findings indicated that RapZ stimulates *glmY* expression to sequester itself when sensing a low GlcN6P level. How can an RBP activate transcription initiation? Transcription of *glmY* can be initiated from two overlapping σ^54^ and σ^70^ promoters. Whereas the latter is weak and unregulated, the stronger σ^54^ promoter is controlled by the TCS QseE/QseF. This system consists of a cytoplasmic membrane bound kinase (QseE) that auto-phosphorylates and subsequently transfers the phosphoryl-group to the response regulator (QseF), which then binds upstream of the *glmY* promoter to activate transcription initiation **(Fig. 2A)**. Notably, we found that RapZ activates QseE/QseF from inside the cell. Two-hybrid assays and SPR indicate that RapZ binds QseE as well as QseF. Concomitantly, RapZ increases auto-phosphorylation of QseE and thereby phospho-transfer to QseF, as revealed by genetic analyses and *in vitro* phosphorylation assays. GlcN6P-bound RapZ is unable to stimulate QseE/QseF. Thus, dependent on metabolite availability RapZ engages in a higher order protein complex to transfer information to the transcription factor controlling its decoy sRNA. The resulting higher GlmY levels sequester RapZ into stable complexes, titrating it away from GlmZ, which then activates *glmS* expression **(Fig. 2A)**. This regulatory mechanism also includes a novel type of feedback loop: GlmY binding interferes with the ability of RapZ to activate QseE/QseF **(Fig. 2A)**. Thus, a sRNA abrogates communication between two signaling proteins. Thereby, *glmY* expression is fine-tuned to the level required to saturate all available RapZ molecules, limiting the response.

**Figure 2:**
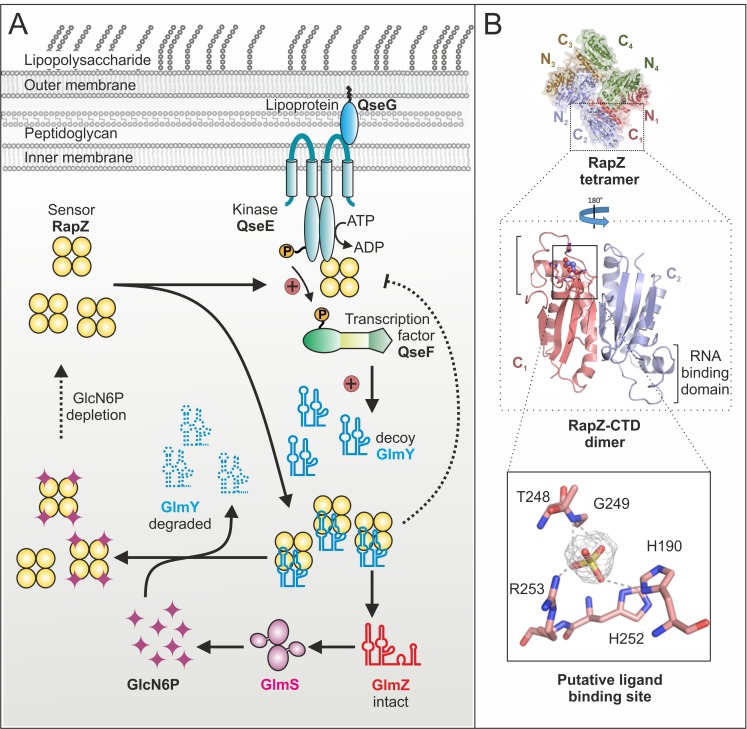
FIGURE 2: GlcN6P sensing and signaling by RapZ. **(A)** Regulatory cascade initiated by RapZ when sensing GlcN6P scarcity. **(B)** Cartoon representation of the X-ray crystal structure of tetrameric RapZ (upper panel). The middle panel shows localization of the RNA binding domain and a putative ligand binding pocket occupied by malonate within the RapZ-CTD swapped dimer. Bottom panel: Close-up view of the residues forming the presumptive GlcN6P binding site with bound sulfate. The cartoons illustrating the RapZ structure are from Gonzalez *et al.*, 2017, *Nucleic Acids Res.* 45(18):10845-10860.

Importantly, GlcN6P not only controls activation of QseE/QseF by RapZ but also governs interaction of RapZ with sRNA GlmY: Once replenished, GlcN6P releases GlmY from RapZ leading to rapid degradation of the sRNA as revealed by EMSA and GlmY half-life determinations **(Fig. 2A)**. Consequently, the accumulation of GlmY observed under GlcN6P starvation conditions results from two distinct activities of RapZ: Up-regulation of *glmY* expression and protection of the produced GlmY through binding. Taken together, our work reveals a multifunctional bacterial sRNA-binding protein whose activity is directly controlled by a metabolite. When GlcN6P is present, RapZ primarily acts as adaptor protein for cleavage of GlmZ by RNase E. When GlcN6P is absent, RapZ communicates with a signaling complex to increase expression of its titrating decoy GlmY. Thus, RapZ regulates and is regulated by one and the same sRNA.

What are the interaction sites used by RapZ to contact its various partners? According to the crystal structure, the RapZ monomer consists of well-separated N-terminal and C-terminal globular domains (NTD/CTD). Four monomers form a domain-swapped dimer of dimers held together through three types of inter-protomer contacts **(Fig. 2B**, top). The tetrameric assembly of RapZ is essential for binding RNase E as well as QseE/QseF and initial data suggest that both domains of RapZ contribute to these interactions. The RapZ-CTD can form a swapped dimer independently, which may bind sRNA GlmY and GlcN6P in a mutually exclusive manner **(Fig. 2B**, middle panel). Intriguingly, the CTD contains a pocket that could accommodate the phosphate moiety of a non-protein ligand and is located in close proximity to the RNA-binding domain **(Fig. 2B**, middle and bottom panel). Thus, it might be speculated that GlcN6P and GlmY compete for binding to the RapZ-CTD. As interaction of RapZ with QseE/QseF is counteracted by GlcN6P as well as sRNA binding, this region may also be part of the surface contacting the TCS. In contrast, the role of the RapZ-NTD is less obvious. The separated RapZ-CTD has higher affinity towards both GlcN6P and GlmY than full-length RapZ. Thus, the NTD might have an auto-inhibitory role. Whether this may be regulated by a different metabolite, as suggested by occurrence of a nucleotide-binding motif in the NTD, remains to be clarified.

Whereas the response of the GlmY/RapZ/GlmZ circuit to GlcN6P starvation is now well understood, it remains unclear how GlcN6P interferes with binding and cleavage of GlmZ by the RapZ/RNase E complex. Intuitively, binding of GlcN6P is expected to stimulate RapZ-mediated decay of GlmZ, but so far there is no evidence that RapZ could discriminate between the two sRNAs. On the other hand, our data indicate that even under normal (i.e. GlcN6P sufficiency) conditions, a fraction of the RapZ molecules remain GlcN6P-free. Interestingly, the putative binding pocket in the RapZ-CTD **(Fig. 2B)** bears homology to the phosphatase loop found in cysteine-dependent protein-tyrosine phosphatases. Perhaps RapZ disengages itself from GlcN6P over time through hydrolysis, allowing it to trigger GlmZ decay when GlcN6P is replenished - a hypothesis that is currently under investigation.

We previously demonstrated that QseE/QseF relies on a third component for function: using its periplasmic domains, QseE interacts with the outer-membrane attached lipoprotein QseG to gain kinase activity **(Fig. 2A)**. Thus, for a fully activated TCS, QseE must be contacted by QseG from outside and by RapZ from inside, forming a large *trans*-envelope signaling complex. Through integration of RapZ, the QseE/QseF TCS is recruited to the GlcN6P starvation signal. Interestingly, QseF also activates expression of *rpoE* encoding the sigma factor orchestrating the cell envelope stress response. It is possible that QseG senses a process in the envelope – perhaps integrity of an amino sugar containing component – to adjust synthesis of cell envelope precursors and envelope repair mechanisms accordingly.

In *E. coli* K-12 *glmS* is the only known target gene regulated by GlmY/GlmZ. In EHEC these sRNAs were recruited to regulate virulence genes, and mutations in QseEGF strongly interfere with virulence in EHEC and other enterobacterial pathogens. Possibly, these pathogens coordinate virulence gene expression with changes in cell envelope synthesis or integrity that may be encountered in the host environment and sensed by QseEGF. In this respect, the GlmY/RapZ/GlmZ circuit and the QseEGF machinery could provide attractive targets for antimicrobial chemotherapy. We previously demonstrated that co-application of the non-metabolizable GlcN6P analog glucosamine-6-sulfate (GlcN6SO_4_) increases the efficacy of GlmS inhibitors such as Nva-FMDP. GlcN6SO_4_ is only weakly active, but understanding how GlcN6P interacts with RapZ may provide a rational basis for designing more potent compounds suppressing GlmY/RapZ/GlmZ. Application of such compounds might not only render cells more susceptible to antibiotics targeting GlmS, but is also expected to suppress virulence of pathogenic *Enterobacteriaceae*.

